# Freezing response-independent facilitation of fear extinction memory in the prefrontal cortex

**DOI:** 10.1038/s41598-017-04335-y

**Published:** 2017-07-13

**Authors:** Jiso Hong, Daesoo Kim

**Affiliations:** Biological Sciences, Korea Advanced Institute of Science and Technology (KAIST), Daejeon, 305-701 Korea

## Abstract

The infralimbic cortex (IL) is known to facilitate the formation of extinction memory through reciprocal interactions with the amygdala, which produces fear responses such as freezing. Thus, whether presynaptic input from the amygdala and post-synaptic output of IL neurons are functionally dissociated in extinction memory formation remains unclear. Here, we demonstrated that photostimulation of IL inputs from BLA did not change freezing responses to conditioned stimuli (CS) during training, but did facilitate extinction memory, measured as a reduction in freezing responses to the CS 1 day later. On the other hand, photostimulation of somata of IL neurons induced an immediate reduction in freezing to CS, but this did not affect extinction memory tested the next day. These results provide *in vivo* evidence for IL-dependent facilitation of extinction memory without post-synaptic modulation of freezing circuits.

## Introduction

The infralimbic cortex (IL) has been implicated in the extinction of conditioned fear responses^1–3^. A mechanism of extinction memory has been proposed based on pioneering studies in rodents employing electrical stimulation^4, 5^ and pharmacological inactivation^6, 7^ of IL neurons, which facilitates or interferes with extinction memory formation. Given that electrical stimulation of the IL reduces freezing response to conditioned stimuli (CS)^5^ and the fact that the amygdala is the neural substrate for freezing responses to CS^8, 9^, it has been thought that the post-synaptic impact of IL neurons on the amygdala is critical for extinction memory. Indeed, connectome studies have shown that efferents of IL neurons innervate inhibitory neurons in the amygdala^10^, explaining how electrical stimulation can reduce the freezing response to CS.

IL neurons are known to receive input from a subset of excitatory neurons in the basolateral amygdala (BLA)^11, 12^. Recent studies suggests that these excitatory BLA neurons are involved in fear extinction^13, 14^. Considering that electrical stimulation activates axon terminals at the stimulation site^15, 16^, afferent presynaptic input from the BLA to the IL could contribute to the extinction memory formation facilitated by electrical stimulation of the IL^17, 18^. A recent study showed that optogenetically induced long-term depression (LTD) of the BLA-IL synapse facilitates extinction memory to CS rather than inhibition, suggesting an inhibitory role of this synapse^19^; thus, the role of presynaptic activity in the IL remains controversial. Here, we optogenetically stimulated input and output circuits separately under the same experimental conditions, then examined effects on freezing responses and retrieval of extinction memory in mice.

## Results

**Figure 1:**
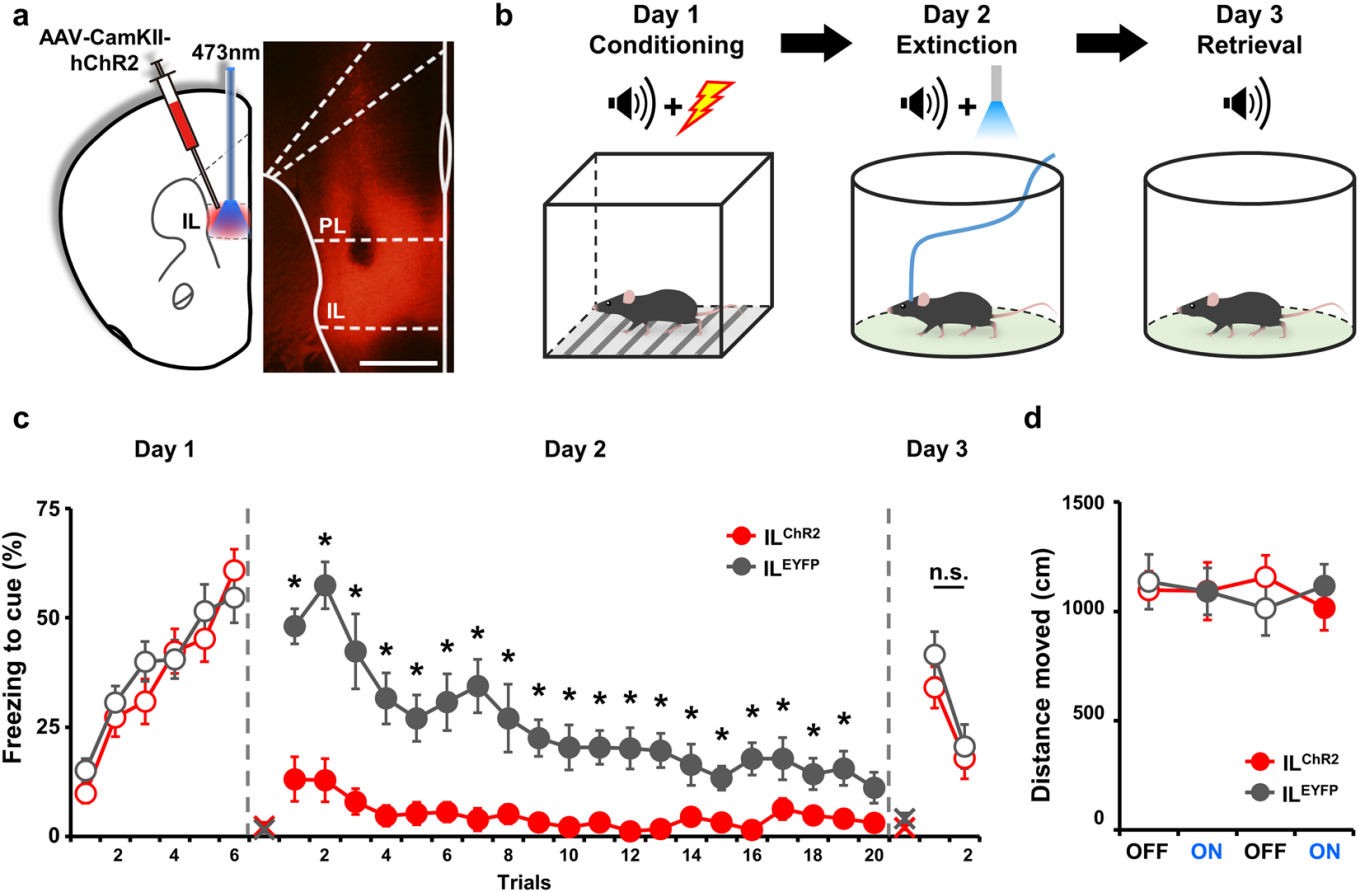
Stimulation of the IL during extinction training decreases freezing without affecting retrieval of extinction memory. (**a**) Schematic representation of AAV2/9-CamKIIa-hChR2(H143R)-mCherry infection and optic fiber placement in the IL. The schematic of the mouse brain is drawn based on the Franklin and Paxinos mouse brain atlas. Scale bar: 500 μm. (**b**) Experimental procedure for extinction with optic stimulation. (**c**) ChR2 stimulation of the IL during extinction training decreased freezing to tone on day 2, but did not facilitate extinction retrieval tested on day 3 (IL^ChR2^, n = 12; IL^EYFP^, n = 10). (**d**) ChR2 stimulation of the IL did not change the locomotor activity of mice in an open field (IL^ChR2^, n = 9; IL^EYFP^, n = 9). Data are presented as means ± s.e.m. Empty circle, absence of optic stimulation; filled circle, presence of optic stimulation; X, baseline freezing level.

To compare input and output circuits in the IL, we separately stimulated each circuit optogenetically, which enables stimulation of specific types of cells or projections^20^. This approach is premised on the idea that previous experiments using electrical stimulation might have stimulated both circuits simultaneously. To stimulate IL somata, we introduced an adeno-associated virus harboring a vector that expresses the light-gated cationic channel, channelrhodopsin (ChR2) under the control of the CamKII promoter (AAV-CamKII-ChR2) into the IL (Fig. 1a, IL^ChR2^). This promoter specifies the expression of ChR2 in pyramidal neurons, except for interneurons^21–24^, and has been used to successfully induce action potentials in response to laser pulses delivered to the medial prefrontal cortex (mPFC)^25^. For illumination of the ChR2-expressing neurons, the optic fiber was located in IL as illustrated in Fig. 1a and Supplementary Fig. S1a. As a control, we used a viral vector expressing enhanced yellow fluorescent protein (EYFP) under the control of the CamKII promoter (IL^EYFP^). On day 1, mice were trained for acquisition of fear memory by coupling a CS (tone) with an unconditioned stimulus (electrical foot shock). On day 2, mice were repetitively exposed to CS coupled with laser stimulation (473 nm, 10 Hz, 20 ms), delivered to the IL by the optic fiber, in a different chamber. The next day, the level of extinction memory was tested in an extinction chamber by measuring the reduction in freezing responses to CS (Fig. 1b). As shown in Fig. 1c, IL^ChR2^ mice showed a greater reduction in freezing responses to CS when coupled with photostimulation compared with control IL^EYFP^ mice (ANOVA group effect: *F*_*1,20*_ = 42.036, *P < 0.001; post hoc: *P < *0.05 for all, except for trial 20 [*P = *0.137]), as described in previous reports using an electrical stimulation paradigm^5^. Surprisingly, however, there was no significant difference in freezing responses between IL^ChR2^ and IL^EYFP^ mice tested the next day without laser stimulation (Fig. 1c; ANOVA group effect: *F*_*1,20*_ = 0.584, *P = *0.453). IL photostimulation could conceivably produce this effect by increasing overall locomotion, which can affect freezing, but a comparison of locomotor activity revealed no significant difference between IL^ChR2^ and IL^EYFP^ mice (Fig. 1d; ANOVA group effect: *F*_*1,16*_ = 0.0000621, *P = *0.994). These data suggest that, although neuronal output from the IL reduces freezing responses, it does not affect retrieval of extinction memory.

**Figure 2:**
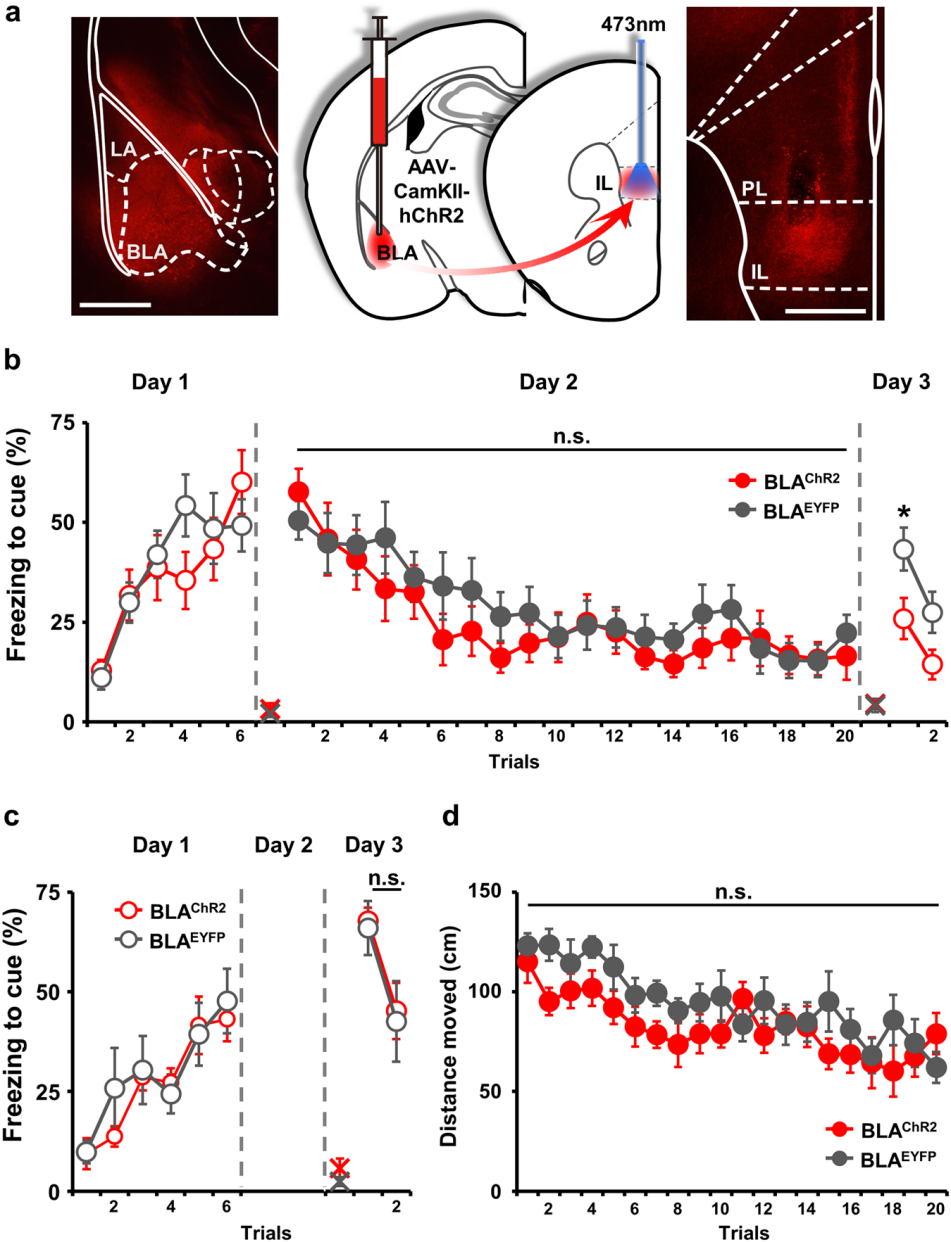
Stimulation of presynaptic input of the BLA during extinction training facilitates extinction memory without affecting freezing responses during extinction training. (**a**) Schematic representation of AAV2/9-CamKIIa-hChR2(H143R)-mCherry infection in the BLA and optic fiber placement in the IL. Scale bar: 500 μm. (**b**) ChR2 stimulation of presynaptic input from the BLA to the IL during extinction training did not affect freezing responses to a tone on day 2, but facilitated extinction retrieval, measured as freezing responses on day 3 (BLA^ChR2^, n = 10; BLA^EYRP^, n = 11). (**c**) Optic stimulation of presynaptic input from the BLA to the IL without extinction training did not affect freezing in retrieval tests (BLA^ChR2^, n = 7; BLA^EYFP^, n = 7). (**d**) Optic stimulation of afferent projections from the BLA to the IL did not alter locomotion (BLA^ChR2^, n = 7; BLA^EYFP^, n = 7). Data are presented as means ± s.e.m. Empty circle, absence of optic stimulation; filled circle, presence of optic stimulation; X, baseline freezing level.

Next, we tested the hypothesis that strengthening of extinction retrieval depends on a presynaptic mechanism, focusing on inputs from the BLA, since this region is known to be involved in fear extinction^13, 14^. To stimulate axonal projections from the BLA to the IL, we delivered a viral vector expressing ChR2 (BLA^ChR2^) or EYFP (BLA^EYFP^) to the BLA (Fig. 2a) and placed an optic fiber in the IL (Supplementary Fig. S1b). To determine whether simulation of projections from the BLA could induce neural activity in the IL, we recorded IL activity *in vivo* following light stimulation in mice expressing ChR2 or EYFP in the BLA (Supplementary Fig. S2a). Blue laser stimulation under the same conditions used in behavioral experiments (10 Hz, 20 ms) successfully induced action potentials in the BLA^ChR2^ group, but failed to affect neural activity in the BLA^EYFP^ group (Supplementary Fig. S2b,c). An analysis of multiunit activity (MUA) showed that the neural activity of the IL was significantly increased by light stimulation in the BLA^ChR2^ group (Supplementary Fig. S2d,f; Wilcoxon Signed Rank test, *P < 0.0001), whereas there was no laser stimulation-dependent change in MUA in the BLA^EYFP^ group (Supplementary Fig. S2e,g; Paired t-test, *t*_*6*_ *= *0.503, *P* = 0.633). During extinction training on day 2, we delivered photostimuli to the IL of BLA^ChR2^ mice to specifically stimulate BLA input to the IL (Fig. 2a). BLA^ChR2^ mice showed no difference in freezing responses to CS compared with BLA^EYFP^ mice in all trials during extinction training (Fig. 2b; ANOVA group effect: *F*_*1,19*_ = 0.413, *P* = 0.528). Interestingly, however, when fear extinction was tested 24 h after extinction, BLA^ChR2^ mice showed significantly decreased freezing responses to CS compared with BLA^EYFP^ mice (Fig. 2b; ANOVA group effect: *F*_*1,19*_ = 5.948, *P = 0.025; post hoc tests: *P = 0.019 for trial 1, and *P* = 0.073 for trial 2). These results support the interpretation that BLA inputs to the IL are critical for the facilitation of extinction memory, which results in stronger retrieval of extinction on the next day.

To test whether stimulation of BLA inputs to the IL actually facilitate extinction memory in a manner that depends on a new association of CS with a ‘safe’ signal, we applied optic stimulation of BLA-IL axonal projection to conditioned mice without tone presentation during day 2 (Supplementary Fig. S1c and Fig. 2c). We found that this CS-free photostimulation caused no differences in fear retrieval between BLA^ChR2^ and BLA^EYFP^ groups (Fig. 2c; ANOVA group effect: *F*_*1,12*_ = 0.0505, *P* = 0.826). Moreover, an analysis of moving distance during laser illumination showed that stimulation of afferent projections from the BLA to the IL had no effect on the locomotor activity of mice (Fig. 2d; ANOVA group effect: *F*_*1,12*_ = 1.828, *P* = 0.201). These results strongly suggest that BLA-to-IL input facilitates extinction memory without affecting post-synaptic modulation of freezing circuits.

## Discussion

Extinction memory is known to be independent of the mechanism that modulates fear memory itself^2^. However, studies have shown that IL neurons modulate extinction memory through their post-synaptic impacts on the amygdala involved in fear memory processing^1, 10^. Our study demonstrates that presynaptic input and postsynaptic output of IL neurons function in a dissociable way: photostimulation of presynaptic inputs from the BLA to the IL facilitates extinction memory (Fig. 2), whereas somatic activation of IL neurons decreases freezing without affecting extinction memory (Fig. 1). These findings strongly suggest that extinction memory can be formed in the IL without affecting post-synaptic modulation of freezing circuits. We confirmed that under our photostimulation conditions, mice showed no difference in locomotor activity compared with control mice in response to CS (Fig. 2b,d) or in an open field test (Fig. 1d), excluding the possibility that the freezing response is affected by a photostimulation-induced change in locomotor behavior.

Our conclusion is consistent with previous reports that extinction of conditioned fear is accompanied by increased field potential responses in the BLA-IL pathway^26^, and that electrical stimulation facilitates extinction memory through enhancement of this projection^5^. In addition, a specific population of BLA neurons known to be activated during acquisition of extinction memory^13^ sends projections to the IL, and optogenetic inhibition of BLA neurons projecting to IL during extinction has been shown to impair retrieval of extinction memory without affecting freezing during illumination^14^, findings that are consistent with our demonstration that stimulation induces facilitation of extinction memory without affecting freezing.

In contrast to previous reports and the conclusions reached here, a recent study reported that the induction of LTD in the BLA-IL synapse by high-frequency photostimulation of the ChR2 variant, ChETA, before extinction learning facilitates the formation of extinction memory, suggesting an inhibitory role of this input^19^. In these latter experiments, however, LTD pretreatment was given before extinction learning and decreased the CS-induced freezing response^19^ before acquisition of extinction memory, suggesting any impact on the freezing circuit mechanism that reduces the fear response itself. However, in our gain-of-function experiment, photostimulation of the BLA-IL synapse was coupled to CS during extinction learning, and this facilitated extinction memory without affecting the freezing-circuit mechanism (Fig. 2b). Thus, the discrepancy between our conclusions and this previous report may reflect differences in experimental conditions, including the timing of optogenetic treatment and the consequent impact on the freezing circuit.

We also demonstrated a post-synaptic impact of IL neurons on the freezing response to CS, even before or during extinction memory learning (Fig. 1c), probably through the well-known projections from the IL to the intercalated nuclei (ICN)^10, 27^, clusters of GABAergic neurons that inhibit freezing outputs from the central amygdala^28, 29^. In a similar vein, high-frequency photostimulation of the BLA-IL synapse to induce LTD^30^ could cause a reduction in freezing through potentiation of this IL-ICN connection, as seen in electrical stimulation of the IL. A previous study reported that the post-synaptic impact of IL neurons is only functional after extinction memory acquisition, as evidenced by the observation that photostimulation of IL neurons has no effect on CS-induced freezing before extinction learning^31^. This discrepancy may be caused by using different mouse strain which can affect fear behavior^32^, however, it remains unclear how the conditions of optogenetic stimulation of IL neurons used in this previous report could bypass the existing post-synaptic impact on the freezing circuit. Consistent with the anatomical connection of the IL to the freezing circuit as well as our hypothesis, previous studies have shown that activation of the IL through electrical^4, 33^, pharmacological^34, 35^, or optogenetic^36^ approaches leads to a significant reduction in freezing responses to CS.

Our results clearly show that extinction memory formation in the IL can occur independent of the postsynaptic effects of the IL on freezing circuits (Fig. 1b). A recent study, however, showed that bilateral photostimulation of ChR2-expressing IL neurons produces the same effect as unilateral electrical stimulation, reducing freezing during extinction memory training and strengthening retrieval of extinction^36^. Our findings suggest that this facilitation of extinction memory may result from activation of presynaptic areas of the IL through activation of callosal inputs from the contralateral IL^18^. Optogenetic stimulation of interhemispheric projections can induce excitatory post-synaptic potentials (EPSPs) in mPFC pyramidal neurons projecting to subcortical regions^37^; thus, simultaneous stimulation of these presynaptic inputs and postsynaptic IL neurons may potentiate the IL^38^ and affect the formation of extinction memory. In addition, a stronger photostimulation (5 mW) was used in the former study than was used here (1 mW), a potentially important difference given that neural and behavioral responses can differ depending on the intensity of light stimulation^39, 40^. Accordingly, stronger photostimulation of IL neurons may induce changes in a broader range of target neurons associated with extinction memory.

The freezing circuit-independent facilitation of extinction memory in the IL may contribute to the fine modulation of extinction memory expression through the freezing circuit. Studies have shown that expression of extinction memory is modulated according to contextual information^3^ encoded by the hippocampus^41, 42^. Given that IL neurons receive inputs from the hippocampus and the amygdala^18^, the integration of various streams of information in the IL would appear to be critical for the expression of extinction memory. Finally, this gating mechanism for extinction memory in the IL could be a relevant target for understanding and treating fear and anxiety disorders, such as post-traumatic stress disorder.

## Materials and Methods

### Subjects

Adult male C57BL/6J mice (10–13 wk old) were used for all experiments. Food and water was available ad libitum under a 12:12 hour light:dark cycle (lights on, 06:00) throughout all experimental procedures. All animal care and experimental procedures were performed in accordance with protocols approved by the Animal Care and Use Committee of Korea Advanced Institute of Science and Technology (KAIST).

### Stereotaxic surgery

Mice were anesthetized with Avertin (2, 2, 2-tribromoethanol; Sigma, USA) and placed on a stereotaxic frame (Neurostar, Germany) for viral infection and implantation of fiber optic cannulae. Two AAV constructs were used for viral expression: AAV2/9-CamKIIa-hChR2(H134R)-mCherry-WPRE-hGH, purchased from University of Pennsylvania Vector Core (https://www.med.upenn.edu/gtp/vectorcore), was used for expression of hChR2 in excitatory pyramidal neurons^21, 22^; and AAV2/5-CamKIIa-eYFP, from the University of North Carolina Vector Core (https://www.med.unc.edu/genetherapy/vectorcore), was used as a control. AAVs were delivered unilaterally to the IL (anteroposterior [AP], + 1.90 mm; mediolateral [ML], ± 0.30 mm; dorsoventral [DV], −2.40 mm) or BLA (AP, −1.30 mm; ML, ± 3.40 mm; DV, −4.10 mm) using a Nanofil syringe with a 33-gauge injection needle (World Precision Instruments, Inc., USA). A monofiber optic cannula (200 μm diameter, 2.5 mm length; Doric Lenses, Canada) was implanted into the IL by affixing it to the skull with Super-Bond (Sun Medical, Japan). After surgery, mice were singly housed to prevent damage of intracranial implants and were allowed 2 wk for recovery and viral expression before behavioral testing.

### Histology

After completion of experiments, the brain was removed, fixed by placing in phosphate-buffered 4% formaldehyde solution for 1–2 d, cut into 60-μm-thick coronal sections using vibratome (Leica VT1200S, Germany), and mounted on slides. Virally mediated expression of fluorescent proteins and the location of optic fibers were confirmed by imaging sections with a fluorescence microscope (Olympus IX41; Olympus, Japan), guided by the Franklin and Paxinos mouse brain atlas^43^. Animals showing off-target optic fiber location or absence of viral expression at the end of the optic fiber were excluded from the analysis.

### Auditory fear conditioning

For auditory fear conditioning (day 1), mice were placed in a standard conditioning chamber (Coulbourn Instruments, USA). After a 3-min habituation, mice received six tones (3000 Hz, 80 dB, 30 s) at 2-min intervals that co-terminated with an electrical shock (0.5 mA, 1 s). On day 2, mice were positioned in an acrylic cylinder (diameter, 23 cm). After a 3-min acclimation period, mice received 20 tones at 40-s intervals to acquire fear extinction. Each tone was coupled with stimulation by blue laser (wavelength, 473 nm) illumination, delivered as 10-Hz, 20-ms pulses. Laser power (~1 mW for IL somata stimulation and ~4 mW for BLA projection stimulation) was measured at the tip of the fiberoptic cannula. For experiments employing stimulation without extinction training, optic stimulation was presented without tones. The next day, mice were placed in the same context as used for extinction training, and retrieval of extinction was tested. After 3 min, two tones were presented at 40-s intervals without optic stimulation. The behavior of mice throughout all procedures was recorded as a video file and analyzed for freezing behavior and locomotor activity using FreezeFrame 3 (Actimetrics, USA) and Ethovision XT (Noldus, Netherlands) software, respectively.

### Open field with optic stimulation

Mice were positioned in the center of 42 × 42 cm acrylic box with white walls and floor. After 15-min acclimation period and a 5-min light-off session, mice received blue laser stimulation (473 nm wavelength, 10 Hz, 20 ms) for 5 min (light-on session). These light off/on sessions were repeated one more time. All mouse behavior was recorded as a video file and analyzed using Ethovision (Noldus, Netherlands).

### Data availability

The datasets generated during and/or analyzed during the current study are available from the corresponding author on reasonable request.

## Electronic supplementary material

Supplementary information

## References

[1] Giustino TF, Maren S (2015). The Role of the Medial Prefrontal Cortex in the Conditioning and Extinction of Fear. Front Behav Neurosci.

[2] Myers KM, Davis M (2007). Mechanisms of fear extinction. Mol Psychiatry.

[3] Orsini CA, Maren S (2012). Neural and cellular mechanisms of fear and extinction memory formation. Neurosci Biobehav Rev.

[4] Vidal-Gonzalez I, Vidal-Gonzalez B, Rauch SL, Quirk GJ (2006). Microstimulation reveals opposing influences of prelimbic and infralimbic cortex on the expression of conditioned fear. Learn Mem.

[5] Milad MR, Quirk GJ (2002). Neurons in medial prefrontal cortex signal memory for fear extinction. Nature.

[6] Morawska MM, Fendt M (2012). The effects of muscimol and AMN082 injections into the medial prefrontal cortex on the expression and extinction of conditioned fear in mice. J Exp Biol.

[7] Sierra-Mercado D, Padilla-Coreano N, Quirk GJ (2011). Dissociable roles of prelimbic and infralimbic cortices, ventral hippocampus, and basolateral amygdala in the expression and extinction of conditioned fear. Neuropsychopharmacology.

[8] Davis M (1997). Neurobiology of fear responses: the role of the amygdala. J Neuropsychiatry Clin Neurosci.

[9] Kim JJ, Jung MW (2006). Neural circuits and mechanisms involved in Pavlovian fear conditioning: a critical review. Neurosci Biobehav Rev.

[10] Pinard CR, Mascagni F, McDonald AJ (2012). Medial prefrontal cortical innervation of the intercalated nuclear region of the amygdala. Neuroscience.

[11] Gabbott PL, Warner TA, Busby SJ (2006). Amygdala input monosynaptically innervates parvalbumin immunoreactive local circuit neurons in rat medial prefrontal cortex. Neuroscience.

[12] Bacon SJ, Headlam AJN, Gabbott PLA, Smith AD (1996). Amygdala input to medial prefrontal cortex (mPFC) in the rat: A light and electron microscope study. Brain Research.

[13] Herry C (2008). Switching on and off fear by distinct neuronal circuits. Nature.

[14] Senn V (2014). Long-range connectivity defines behavioral specificity of amygdala neurons. Neuron.

[15] Nowak LG, Bullier J (1998). Axons, but not cell bodies, are activated by electrical stimulation in cortical gray matterII. Evidence from selective inactivation of cell bodies and axon initial segments. Experimental Brain Research.

[16] Kringelbach ML, Jenkinson N, Owen SLF, Aziz TZ (2007). Translational principles of deep brain stimulation. Nat Rev Neurosci.

[17] Vertes RP (2004). Differential projections of the infralimbic and prelimbic cortex in the rat. Synapse.

[18] Hoover WB, Vertes RP (2007). Anatomical analysis of afferent projections to the medial prefrontal cortex in the rat. Brain Struct Funct.

[19] Klavir O, Prigge M, Sarel A, Paz R, Yizhar O (2017). Manipulating fear associations via optogenetic modulation of amygdala inputs to prefrontal cortex. Nat Neurosci.

[20] Yizhar O, Fenno LE, Davidson TJ, Mogri M, Deisseroth K (2011). Optogenetics in neural systems. Neuron.

[21] Liu XB, Jones EG (1996). Localization of alpha type II calcium calmodulin-dependent protein kinase at glutamatergic but not gamma-aminobutyric acid (GABAergic) synapses in thalamus and cerebral cortex. Proc Natl Acad Sci USA.

[22] Van den Oever MC (2013). Ventromedial Prefrontal Cortex Pyramidal Cells Have a Temporal Dynamic Role in Recall and Extinction of Cocaine-Associated Memory. The Journal of Neuroscience.

[23] Johansen JP (2010). Optical activation of lateral amygdala pyramidal cells instructs associative fear learning. Proceedings of the National Academy of Sciences.

[24] Goshen I (2011). Dynamics of retrieval strategies for remote memories. Cell.

[25] Lee E (2015). Left brain cortical activity modulates stress effects on social behavior. Sci Rep.

[26] Vouimba RM, Maroun M (2011). Learning-induced changes in mPFC-BLA connections after fear conditioning, extinction, and reinstatement of fear. Neuropsychopharmacology.

[27] Berretta S, Pantazopoulos H, Caldera M, Pantazopoulos P, ParÉ D (2005). infralimbic cortex activation increases c-fos expression in intercalated neurons of the amygdala. Neuroscience.

[28] Paré, D. & Smith, Y. The intercalated cell masses project to the central and medial nuclei of the amygdala in cats. *Neuroscience* **57**, 1077–1090, doi:http://dx.doi.org/10.1016/0306-4522(93)90050-P (1993). 10.1016/0306-4522(93)90050-p 8309544

[29] Likhtik, E., Popa, D., Apergis-Schoute, J., Fidacaro, G. A. & Pare, D. Amygdala intercalated neurons are required for expression of fear extinction. *Nature* **454**, 642–645, doi:http://www.nature.com/nature/journal/v454/n7204/suppinfo/nature07167_S1.html (2008). 10.1038/nature07167 PMC2528060 18615014

[30] Amir A, Amano T, Pare D (2011). Physiological identification and infralimbic responsiveness of rat intercalated amygdala neurons. Journal of Neurophysiology.

[31] Kim HS, Cho HY, Augustine GJ, Han JH (2016). Selective Control of Fear Expression by Optogenetic Manipulation of Infralimbic Cortex after Extinction. Neuropsychopharmacology.

[32] Camp M (2009). Impaired Pavlovian fear extinction is a common phenotype across genetic lineages of the 129 inbred mouse strain. Genes, Brain and Behavior.

[33] Milad MR, Quirk GJ (2002). Neurons in medial prefrontal cortex signal memory for fear extinction. Nature.

[34] Thompson BM (2010). Activation of the infralimbic cortex in a fear context enhances extinction learning. Learn Mem.

[35] Chang C-h, Maren S (2011). Medial prefrontal cortex activation facilitates re-extinction of fear in rats. Learning & Memory.

[36] Do-Monte FH, Manzano-Nieves G, Quinones-Laracuente K, Ramos-Medina L, Quirk GJ (2015). Revisiting the role of infralimbic cortex in fear extinction with optogenetics. J Neurosci.

[37] Lee, A T. *et al*. Pyramidal Neurons in Prefrontal Cortex Receive Subtype-Specific Forms of Excitation and Inhibition. *Neuron* **81**, 61-68, doi:http://dx.doi.org/10.1016/j.neuron.2013.10.031 (2014). 10.1016/j.neuron.2013.10.031 PMC3947199 24361076

[38] Feldman, D E. The Spike-Timing Dependence of Plasticity. *Neuron* **75**, 556–571, doi:http://dx.doi.org/10.1016/j.neuron.2012.08.001 (2012). 10.1016/j.neuron.2012.08.001 PMC3431193 22920249

[39] Lee H (2014). Scalable control of mounting and attack by Esr1 + neurons in the ventromedial hypothalamus. Nature.

[40] Lin, D. *et al*. Functional identification of an aggression locus in the mouse hypothalamus. *Nature* **470**, 221–226, doi:http://www.nature.com/nature/journal/v470/n7333/abs/10.1038-nature09736-unlocked.html#supplementary-information (2011). 10.1038/nature09736 PMC3075820 21307935

[41] Bouton ME (2004). Context and behavioral processes in extinction. Learn Mem.

[42] Corcoran KA, Desmond TJ, Frey KA, Maren S (2005). Hippocampal inactivation disrupts the acquisition and contextual encoding of fear extinction. J Neurosci.

[43] Paxinos, G. & Franklin, K. B. *The mouse brain in stereotaxic coordinates*. (Gulf Professional Publishing, 2004).

