# Supplementary material for: Freezing response-independent facilitation of fear extinction memory in the prefrontal cortex
Source: Sci Rep. 2017 Jul 13;7:5363. doi: 10.1038/s41598-017-04335-y (PMC5509670; doi:10.1038/s41598-017-04335-y)

291 Daehak-ro, Yuseong-gu, Daejeon
305-701, Republic of Korea
Tel: +82-42-350-2639
Fax: +82-42-350-2631
https:// http://bs.kaist.ac.kr/~brain

Freezing response-independent facilitation of fear extinction memory in the prefrontal cortex

Jiso Hong[1] and Daesoo Kim[1*]

[1]Biological Sciences, Korea Advanced Institute of Science and Technology (KAIST), Daejeon, 305-701, Korea

*Correspondence to:

Daesoo Kim (daesoo@kaist.ac.kr)

Associate Professor

Behavioral Genetics Lab

Department of Biological Sciences

Korea Advanced Institute of Science & Technology (KAIST)

291 Daehak-ro, Yuseong-gu

Daejeon 34141

Office: 82-42-350-2639

291 Daehak-ro, Yuseong-gu, Daejeon
305-701, Republic of Korea
Tel: +82-42-350-2639
Fax: +82-42-350-2631
https:// http://bs.kaist.ac.kr/~brain

Supplementary figures and legends

Supplementary figure S1. Location of optic fibers a, Placement of optic fibers in IL^{ChR2} and IL^{EYFP}. Numbers are the distance from the bregma on anterial-posterial axis. The schematic of the mouse brain is drawn based on the Franklin and Paxinos mouse brain atlas. **b,** Placement of optic fibers in BLA^{ChR2} and BLA^{EYFP} in extinction experiments (Fig. 2b). **c,** Placement of optic fibers in BLA^{ChR2} and BLA^{EYFP} in no-extinction experiments (Fig. 2c).

Supplementary figure S2. In vivo recording of IL activity induced by optogenetic stimulation of BLA-IL projection. a, Schematic representation of AAV2/9.CamKII-hChR2(H143R)-mCherry infection in the BLA (left panel) and placement of recording electrode in the IL (right panel). Yellow arrow indicates the track of electrode marked by DiI. Scale bar: 500μm. **b,c,** The representative trace of multiunit recording of ChR2 group (left) and EYFP group (right) following 10Hz, 20msec pulse stimulation with a 473nm laser. **d,e,** Perisitimulus histogram of multi-unit activity (MUA) count per 1 msec bin recorded from BLA^{ChR2} (d, small graph shows MUA activity in 0-100 counts/bin range.) and BLA^{EYFP} (e) during 10Hz optic stimulation. Blue transparent rectangle indicates 20msec laser pulse. Shaded areas represent s.e.m. (BLA^{ChR2} group, n = 21; BLA^{EYFP} group, n = 6). **f,g,** Change in MUA per 20 msec bin by laser stimulation in BLA^{ChR2} (e) and BLA^{EYFP} group (f). BLA^{ChR2} No light vs. Light $^{*}P < 0.0001$, Wilcoxon Signed Rank Test; BLA^{EYFP} No light vs. Light $t_6 = 0.503$ $P = 0.633$, paired t-test. Data are represented as means ± s.e.m. Empty bar, absence of laser pulse; filled bar, presence of laser pulse.

291 Daehak-ro, Yuseong-gu, Daejeon
305-701, Republic of Korea
Tel: +82-42-350-2639
Fax: +82-42-350-2631
https:// http://bs.kaist.ac.kr/~brain

Supplementary figure S1.

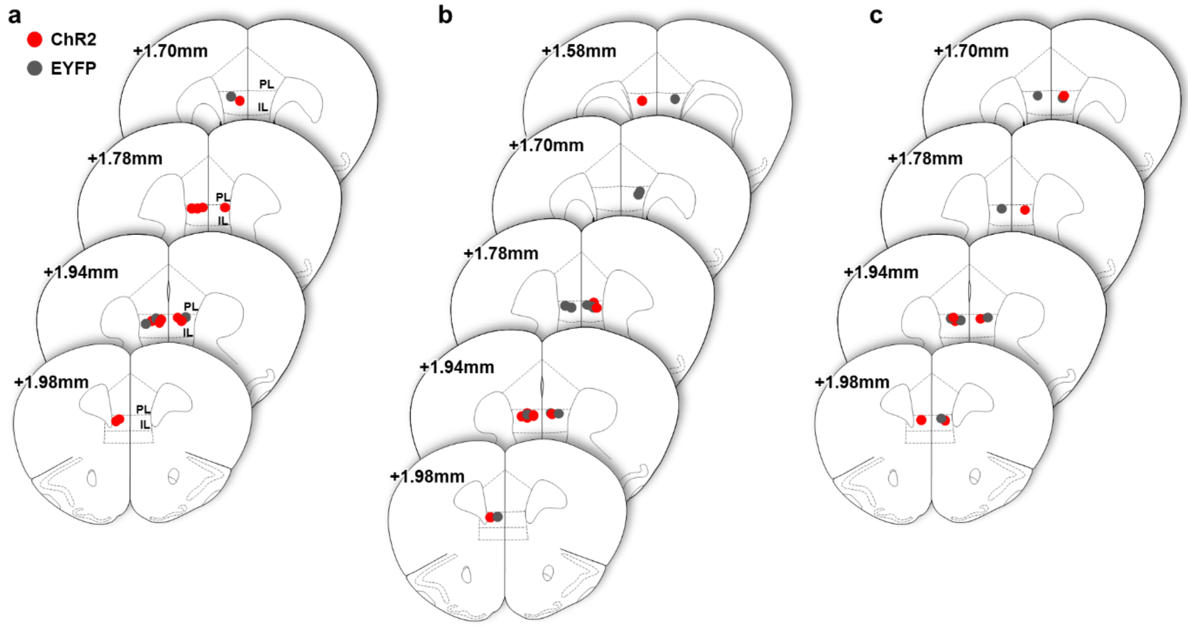

291 Daehak-ro, Yuseong-gu, Daejeon
305-701, Republic of Korea
Tel: +82-42-350-2639
Fax: +82-42-350-2631
https:// http://bs.kaist.ac.kr/~brain

Supplementary figure S2.

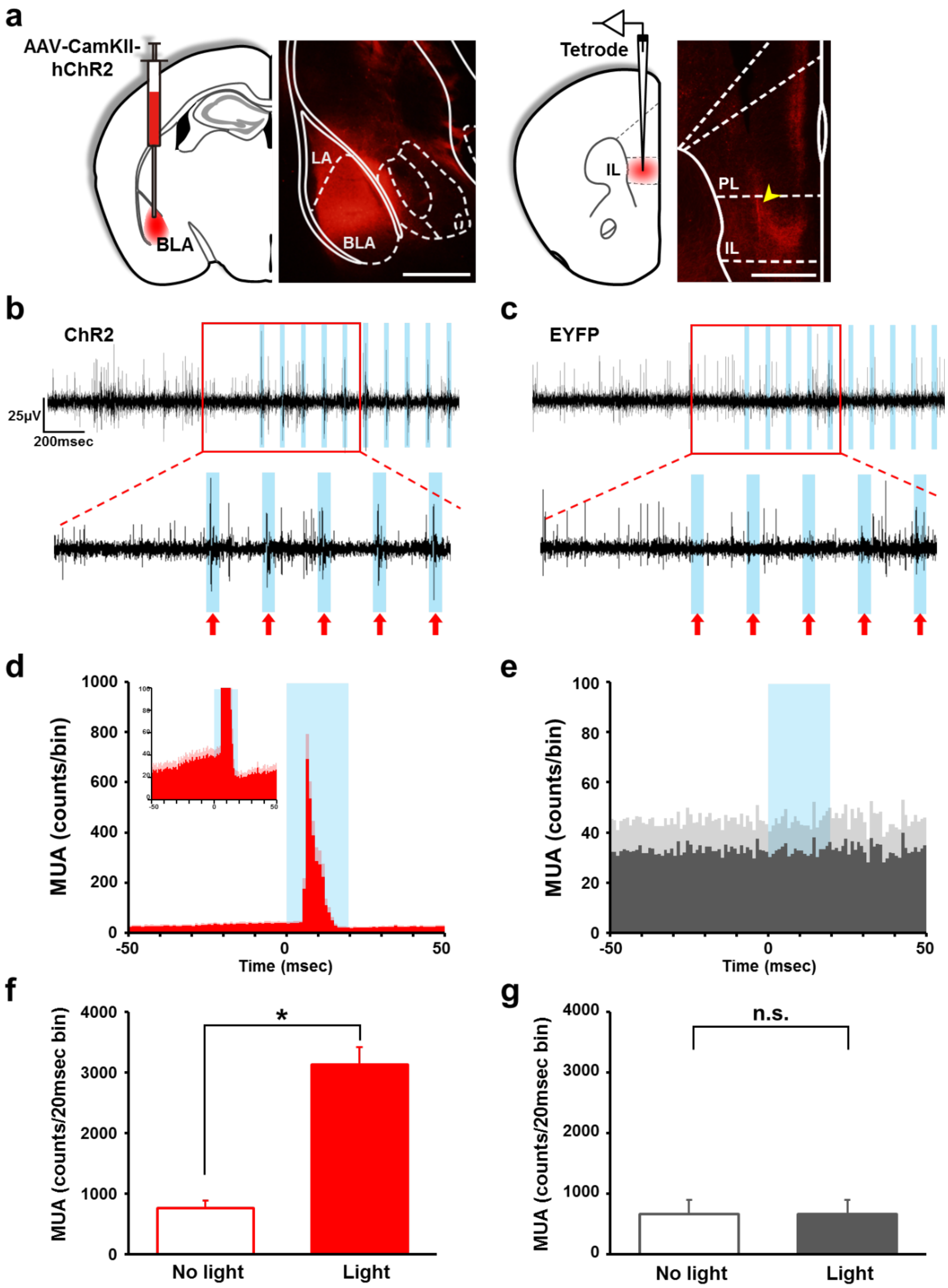

Supplement: Supplementary file 1 — Supplementary information [file 41598_2017_4335_MOESM1_ESM.pdf]
